# Supplementary material for: The ELECTRA Trial: Approach to Contemporary Challenges in the Development and Implementation of Double-Blinded, Randomised, Controlled Clinical Trials in Low-Volume High-Complexity Surgical Oncology
Source: Cancers (Basel). 2025 Jan 21;17(3):341. doi: 10.3390/cancers17030341 (PMC11816246; doi:10.3390/cancers17030341)
Supplement: Supplementary file 1 [file cancers-17-00341-s001.zip › cancers-3419321-File S1.pdf]

Supplementary file S1

Contents:

1. Pathology standards: specimen handling and reporting of pelvic exenteration specimens for colorectal cancer in the ELECTRA trial; page 1

2. Radiology standards: MRI staging and reporting of patients recruited to the ELECTRA trial; page 8

1. Pathology standards: specimen handling and reporting of pelvic exenteration specimens for colorectal cancer in the ELECTRA trial

1.1 Overview

The most important indicator of the effectiveness of IOERT is the incidence of IOERT treated field local relapse in patients with a close or microscopically involved resection margin. Consequently, an important objective of the ELECTRA feasibility trial is to establish rigorous and standardised criteria for the preparation and evaluation of the resected pelvic exenteration specimen. In addition, in any subsequent late phase multi-centre follow on trial to ELECTRA, standardisation and quality control in the pathological approach to the resected tumour specimen will be critical, and the nominated institutions GI histopathologist will be asked to attend physical or remote educational training days.

1.2 Background

The cancer datasets published by The Royal College of Pathologists (RCPath) of the UK are a combination of textual guidance, educational information, and reporting proformas. The datasets enable pathologists to grade and stage cancers in an accurate, consistent manner in compliance with international standards and provide prognostic information thereby allowing clinicians to provide a high standard of care for patients and appropriate management for specific clinical circumstances.

Pelvic exenteration may be defined as a radical and extreme surgical procedure for the *en bloc* removal of internal abdomino-pelvic organs and tissues contiguously involved by locally advanced or locally recurrent cancers. Pelvic exenteration has evolved in recent decades and specimens sent to histopathology often include different abdomino-pelvic organs, major neurovascular structures, and often bony elements and in whom radical surgery often forms part of a multi-modality approach to cancer therapy.

Outcome in these cases is strongly dependant on the completeness of tumour excision, with the most optimal outcomes achieved in patients with a clear resection margin (R0), while those with clear but very narrow margins, or microscopically involved margins (R1), or gross macroscopic residual disease (R2) have significantly poorer results and survival.

Consequently, the pathology reporting of such specimens is a strong predictor of outcome and may form part of the process for determining at specialist cancer multidisciplinary team meetings what subsequent management patients should undergo. In addition, the nature of these surgical specimens is that they may represent some of the most complex specimens sent to pathology departments at present. Nevertheless, there is no specific guideline established nationally at present for their handling, processing, and subsequent reporting and the current dataset for rectal cancer does not cover recurrent rectal cancer. Consequently, while surgery for recurrent rectal cancer clearly takes place, it is assumed therefore that most units adapt their reports using the standard rectal cancer dataset.

The present document therefore sets out a proposed specimen handling and reporting strategy for complex exenterative multi-visceral specimens.

This document builds on the revised national datasets for colorectal cancer and is intended to provide a road map for pathological specimen handling for associated clinical trials in this space.

1.3 Specific notes

For ease of use, all recommendations present and described in the Royal College of Pathologists document entitled “Dataset for histopathological reporting of colorectal cancer” version 4 dated September 2018 have been adopted and are taken as given for the present document. The T, N and M stage changes and refinements of TNM 8 have been adopted. It is recommended that a clear detailed history is provided on any specimen forms sent with such specimens to detail the previous neoadjuvant treatments as well as any prior surgery the patient may have had.

1.4 Preparation of specimens before dissection

Ideally, specimens should be received fresh and unopened as soon as possible after surgical resection but, in practice, and especially given the duration of operating time for pelvic exenteration specimens, the vast majority are received in formalin fixative.

If not delivered fresh to the laboratory, the specimen should typically be placed unopened in a large volume of formalin fixative. Even if a significant delay (>24hours) is anticipated prior to handling in the laboratory, for example when surgery is performed at the weekend, the specimen should be placed in formalin while awaiting collection to minimise autolysis.

Typically, such large thick specimens will require a greater period of time for the fixative to reach the centre of the specimen. As a result, early cautious opening of hollow organs such as the bowel or bladder, without interfering with potential tumor margins, to allow better penetration of the fixative is advantageous. At this stage a foam or wick may be passed into the lumen of the individual hollow organ to aid fixative permeation (figure S1).

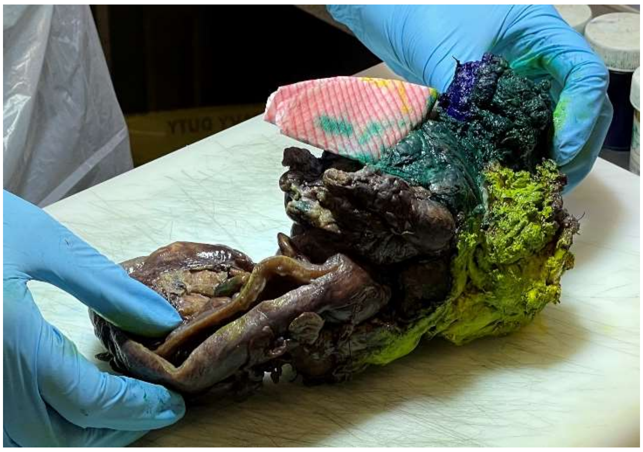

Figure S1: pelvic exenteration specimen that has been formalin fixed and painted with rectosigmoid lumen opened without interference with any cancer margins and wick inserted into bladder lumen to better enable fixative permeation.

1.5 Specimen handling and block selection

Figure S2 shows examples of fresh and fixed pelvic exenteration specimens that may be encountered in ELECTRA. From these figures, it can be seen that especially after fixation, and if insufficient information is provided on pathology forms, the exact delineation of anatomy by the pathology team, particularly if anatomy is unmarked or not assessed in the presence of the surgeon, can be challenging. Consequently the initial specimen handling and cut up is an important process to have standardised.

For descriptive purposes and subsequent management, specimens are sub-categorised for macroscopic handling into 2 categories, those containing bone and those not containing bone. Subsequent microscopic analysis is similar.

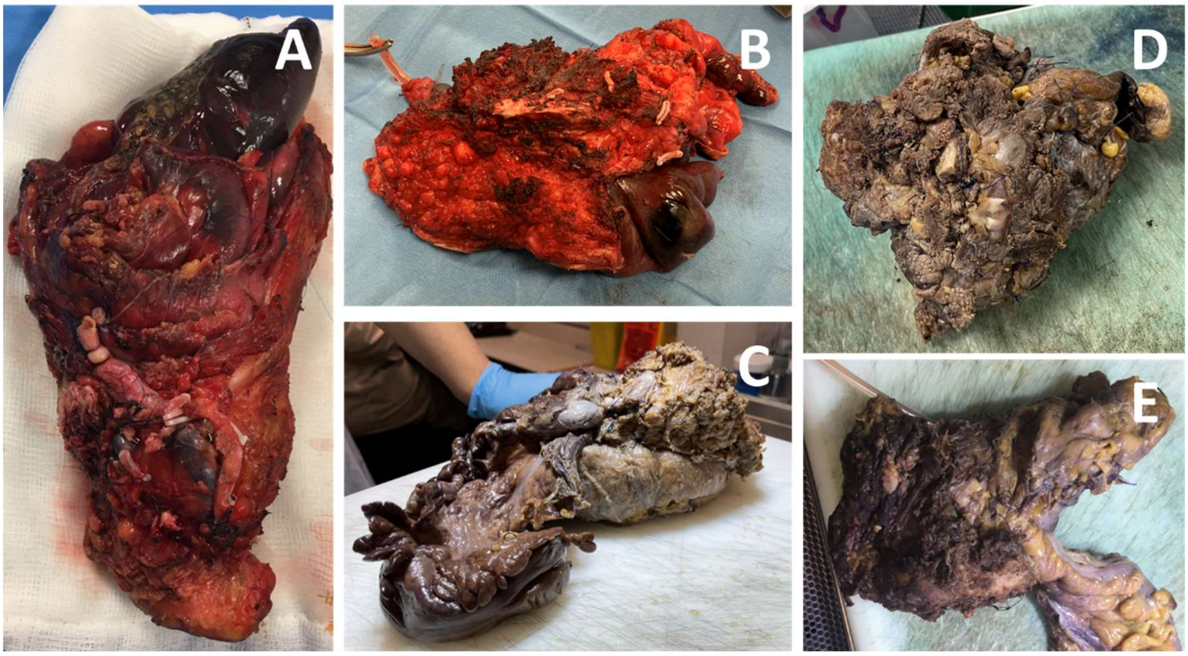

Figure S2: Diverse group of fresh post-operative and formalin fixed specimens after exenterative surgery that may be encountered as part of the ELECTRA trial. Especially after fixation, delineation of anatomy by the pathology team, especially if anatomy is unmarked or not done in the presence of the surgeon, can be difficult. (A) Total supralevator pelvic exenteration with unilateral en bloc sidewall resection and en bloc S1 and S2 high subcortical sacrectomy for LARC. (B) Total pelvic exenteration with en bloc distal sacrectomy, en bloc resection of the ischial spine and loops of adherent small bowel for LRRC. (C) Total pelvic exenteration with en bloc distal sacrectomy, en bloc resection of pelvic sidewall and unilateral ischial spine and sciatic nerve for LRRC. (D) Total pelvic exenteration with en bloc resection of distal sacrum, ischial spine, and pelvic sidewall for LRRC. (E) Total pelvic exenteration with S2 level sacrectomy for LRRC.

1.6 Macroscopic assessments:

1.6.1 Specimens not containing bone

From figure S2, it can be seen that orientation and identification of relevant structures after fixation can be challenging. Different muscles attached to the specimen (e.g. obturator internus, piriformis, gluteal muscles, levator plate muscles) or major blood vessels and any bony elements may look remarkably similar and be unfamiliar to the reporting pathologists in a non-orientated specimen, and so a joint evaluation can be helpful.

Consequently, it is recommended that the operating surgeon or a member of the surgical team either orientates the specimen with the reporting pathologist at a mutually suitable time, or provides sufficient orientation e.g. marking with sutures and or painting of the specimen in the theatre suite (e.g. figure S3). This will need to be accompanied by sufficient explanatory notes on the pathology request form.

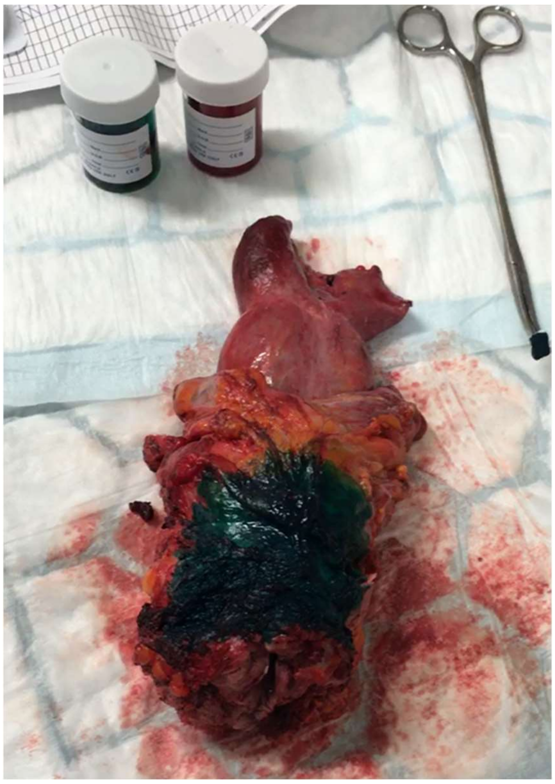

Figure S3: Specimen post exenteration with the margin of concern painted in theatre by the operating surgeon using a selection of paints that can be kept in the theatre suite.

In addition, whereas for conventional colorectal specimens (anterior resection or APE specimen), an external examination may enable location of the tumour, this is usually not the case for exenterative specimens.
If, however there is obvious tumour exposure at a non-peritonealised surface then this should be recorded.
At this stage the macroscopic data items should be recorded.
It is recommended that after the initial joint inspection of the intact and fixed surgical specimen, the specimen should be photographed and subsequently the circumferential (non-peritonealised) surgical resection margin in the vicinity of the tumour should be painted.
Different colours may be used to highlight different margins especially for tumours advanced in different directions. This then provides additional information and feedback for the surgical and radiology teams regarding which margin may have been inadvertently compromised.
After painting, the specimen is sprayed with acetic acid and dabbed dry. At this stage the specimen is ready for cutting with a sharp blade in the predicted vicinity of the tumour.
Sectioning should typically take place in the anatomically horizontal plane to mirror the MRI scans and surgical planning.
Sections should be taken typically at 3-4 mm intervals and slices laid out sequentially for inspection and photography, enabling a permanent record of the macroscopic appearances to be kept for presentation and MDT review.
At this stage inspection will identify areas of concern for sampling using conventional or megablocks and the distance to the nearest CRM will be notable.
These images will also be useful to correlate with preoperative radiological appearances that were used to plan the surgical planes and provide useful feedback to the surgical and radiology teams. For the purposes of clinical trials, it is recommended that the photography of each tumour should be forwarded to the trials office.

It is important to note that in some patients there may be multi-focal recurrences and the nearest CRM may not be the one for the most prominent tumour.

1.6.2 Specimens containing bone

For specimens containing bone, additional steps may be required. Specimen orientation and or painting prior to any dissection is important and communication and or joint assessment of the tumour by surgeon and pathologist is strongly recommended.

The initial painting process can be similar to that for specimens without bone.

Broadly, there are two options for specimen handling in this situation.

Firstly, after orientation, the surgeon and pathologist may elect to cut the section of pelvic bone away from the main specimen using a sharp scalpel and to mark/paint the inside margin of the cut away bone for future identification and awareness that this margin contained an inner table of bone (figure S4).

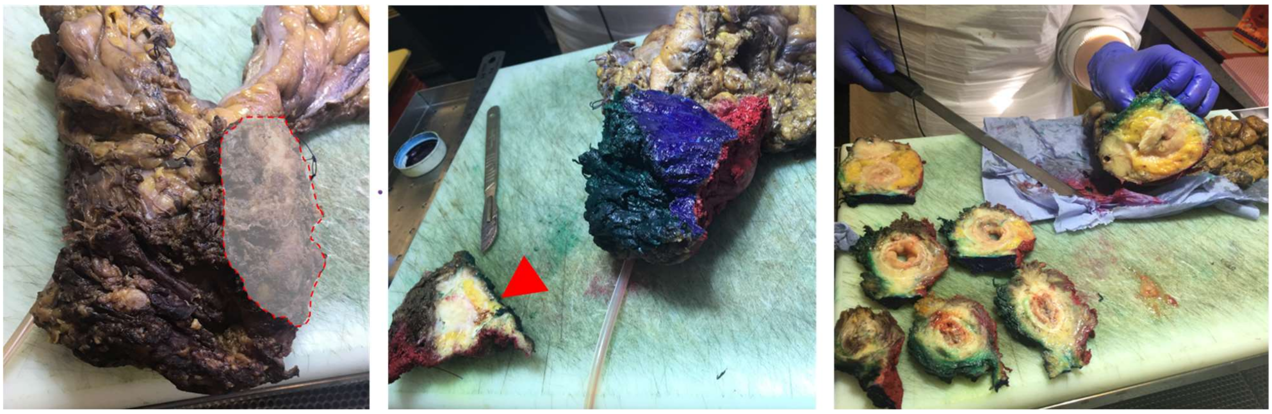

Figure S4: Illustrative images of a patient who has had IOERT to the pelvic sidewall following an exenterative operation for LARC. The example shown represents an infralevator total pelvic exenteration with en bloc S3 level sacrectomy and en bloc pelvic sidewall resection. The left panel shows the post-fixation specimen with the sacrum attached prior to inking (grey with red edge). The middle panel shows that this is then inked jointly by surgeon and pathologist and bony segments excised to enable sectioning (red arrow). In the middle panel the extent of cancer creeping cephalad up the sacrum can be seen. In the right panel, the specimen is bread-loafed prior to finer sectioning.

Secondly, and if possible, the surgeon and pathologist may elect to use a pathology department diamond band saw (e.g the EXAKT system; figures S5) to cut the whole specimen.

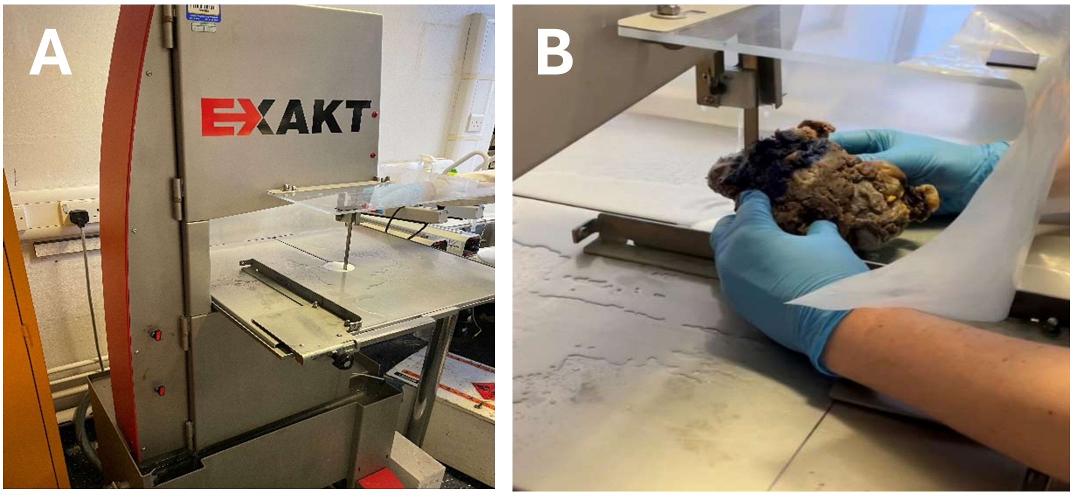

Figure S5: (A) Pathology department band saw being used to cut a pelvic exenteration specimen containing sacrum and ischial spine (B) which have been taken en bloc as part of the extended margin.

This process allows a macroscopic examination of the pathology specimen by the surgeon and pathologist in planes recognisable from the pre-operative MRI surgical planning discussions. It also provides good anatomical feedback to surgeons and radiologists about the closest margin, and how their preoperative and operative steps may have affected the resection margin.
Finally, it also identifies, for pathologists, the anatomical zone to most sample for clarity on the nearest CRM and the R stage of the tumour (figures S6 and S7).

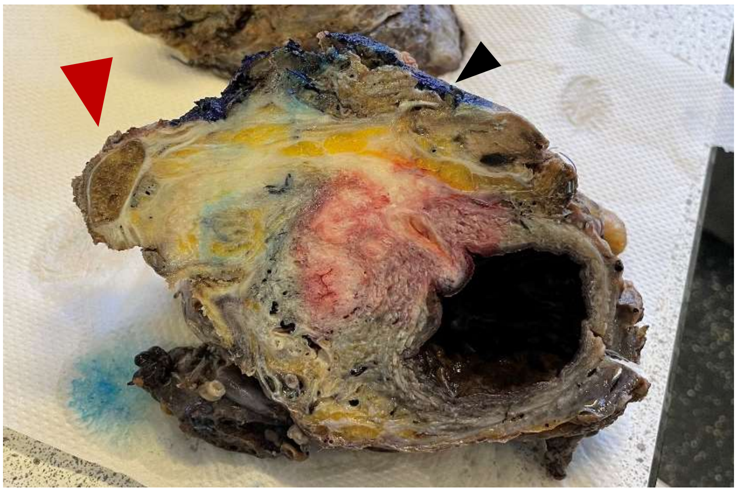

Figure S6: Example of pelvic exenteration specimen with en bloc ischial spine and pelvic sidewall resection after cutting with diamond band saw. Red arrow head marks the cut sacrum and black arrow head marks the ischial spine. The blue painted margin of the sidewall (which in this specimen was the most concerning margin and the site of IOERT can be seen.

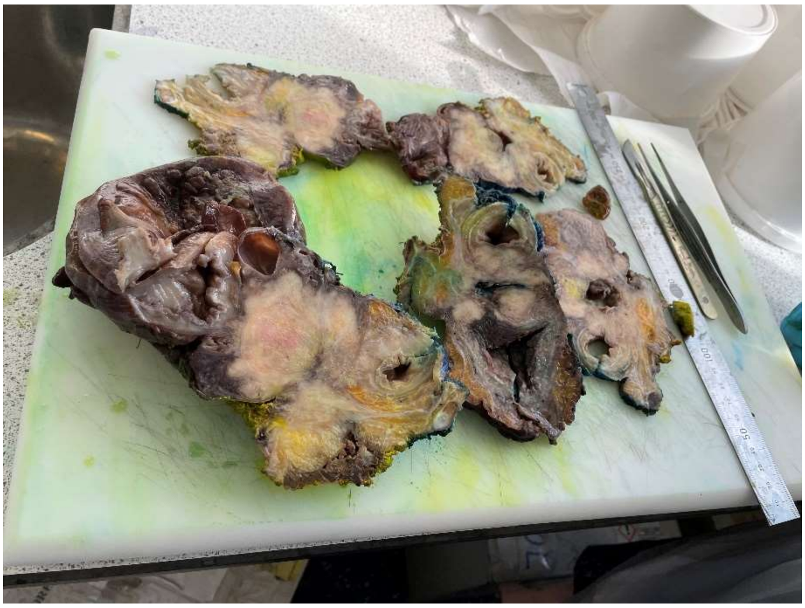

Figure S7: Multiple sections can be cut at 3-4 mm intervals and then photographed before taking blocks.

1.7 Microscopic assessments:

After the above cut up, the area in which the tumour spreads closest to the resection margin should be identifiable. Large blocks should be taken from this area and any area where the tumour extends to within 5 mm from the margin. Photographs of the closest margin should be taken and recorded for the purposes of the trial. A measurement of tumour at 1mm or less from a resection margin is recorded as a microscopically involved margin or R1 resection.

All further assessments e.g. of EMVI, regression grading, quality of the mesorectum (where present) should be recorded as per the latest RCPath guidelines.

The closest aspect of the tumour to the CRM should be sampled. A record should be kept if possible of whether iliac vessel branches and or sacral nerve roots are involved with tumour or abutted by tumour. The proximal and distal relationships of the tumour should also be recorded.

All lymph nodes or tumour deposits identified should be subjected to microscopy.

Peritoneal involvement should be clearly recorded.

In tumours that have received neoadjuvant treatment, evaluation of the extent of regression and or the presence of complete response is identical to conventional colorectal specimens. Evidence of tumour regression especially at the nearest CRM should be reported and a TRS grade given and recorded as part of the ELECTRA trial.

Any lympho-vascular or perineural invasion should be documented.

The greatest distance of direct spread outside the muscularis propria should be recorded.

2. Radiology standards: MRI staging and reporting of patients recruited to the ELECTRA trial

2.1 Overview

Patient selection for extended margin surgery in the pelvis and the application of IOERT is determined by MRI staging, which therefore is a key indicator of the eligibility for recruitment to the ELECTRA trial.
However, the exact methods and protocols for such staging are not standardised in the UK or internationally, and such variation greatly affects the ability to plan to use IOERT or the ability to interpret the results of such an intervention.

Examples of optimal and suboptimal MRI staging of advanced pelvic tumours are shown in figure S8 below. In the left panel, although a locally advanced mid rectal cancer is present however the planning of margins and the extent of sidewall involvement are unclear. On the right panel, the imaging shows a much sharper definition of the anatomy and allows better planning showing that the case shown is not eligible for the ELECTRA trial as the tumour extension does not involve the pelvic sidewall.

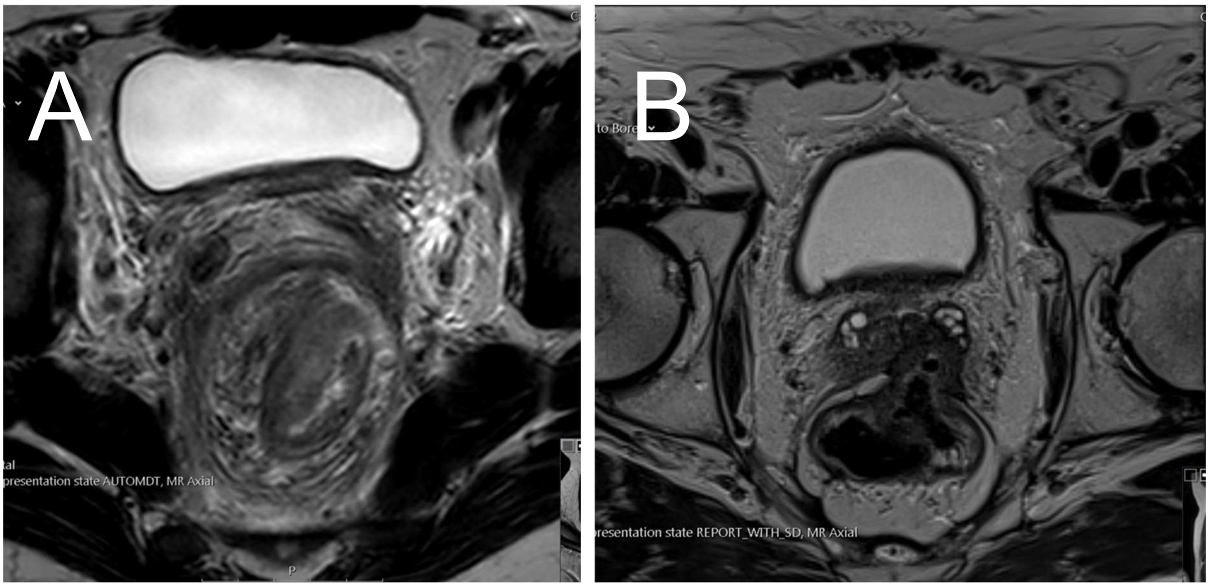

Figure S8: (A) Poor resolution T2 weighted axial MRI image which is sub-optimal for operative planning. (B) High resolution better quality image.

Consequently, an important objective of the ELECTRA feasibility trial is to establish rigorous and standardised criteria for the preoperative imaging of patients for extended margin surgery.
The following MRI protocols should therefore be applied in the feasibility stage of the ELECTRA trial, and for descriptive purposes are subdivided into protocols for where the rectum is present (e.g. LARC or LRRC after previous anterior resection or Hartmanns type operations) vs a protocol for when the rectum is not present (e.g. LRRC after previous APER):

2.2 Rectal MRI Protocols

(Slices/Gap%/Thickness, FOV)

Complex – rectum in situ: Supervised LARC or LRRC

- Sagittal SFOV high resolution T2 30/20/3, 230/121.9%

- Axial T1 whole pelvis 40/20/5, 380
- Axial True LFOV high resolution T2 30/20/4, 380
- Coronal Oblique SFOV high resolution T2 20/10/3, 200
- Axial Oblique SFOV high resolution T2 20/10/3, 200
- DWI axial whole pelvis 40/20/5, 380

Review and perform if needed:

- Sagittal T1 30/20/3, 250
- Post contrast Axial T1 30/20/3, 250

(45 MINS)

Complex – rectum removed: Supervised LRRC

- Sagittal high resolution T2 – sidewall to sidewall 45/10/4, 230/121.9%
- Axial T1 whole pelvis 40/20/5, 380
- Axial True high resolution T2 – sigmoid to perineum 30/20/4, 380
- Coronal True high resolution T2 50/10/3, 200
- DWI axial whole pelvis 40/20/5, 380

Review and perform if needed:

- Sagittal T1 45/10/4, 250
- Post contrast Axial T1 45/10/4, 250

(45 MINS)
