# Supplementary material for: The ELECTRA Trial: Approach to Contemporary Challenges in the Development and Implementation of Double-Blinded, Randomised, Controlled Clinical Trials in Low-Volume High-Complexity Surgical Oncology
Source: Cancers (Basel). 2025 Jan 21;17(3):341. doi: 10.3390/cancers17030341 (PMC11816246; doi:10.3390/cancers17030341)
Supplement: Supplementary file 1 [file cancers-17-00341-s001.zip › cancers-3419321-File S2.pdf]

Supplementary file S2

ELECTRA Trial documentation:

The full ELECTRA trial protocol however is available in detail elsewhere (www.southampton.ac.uk/ctu/trialportfolio/listoftrials/electra.page). For the purposes of this manuscript, summaries are provided below of pertinent sections however.

ELIGIBILITY

Inclusion criteria

- Aged ≥16 years
- Non-metastatic/oligo-metastatic (up to 3 lesions from 2 sites predicted to be radically treatable) – LARC or LRRC involving the posterior or lateral compartments of the pelvis and predicted to be resectable but with close or involved margins from MRI as determined by a specialist MDT (sMDT)
- Colorectal sMDT review with experience in pelvic exenteration, which has proposed IntraOperative Electron Radiotherapy (IOERT) as an option for treatment
- Patient suitable for IOERT as a component of treatment in the view of the responsible Clinical Oncologist
- Performance status ≤1 as defined by the Eastern Cooperative Oncology Group (ECOG)
- Deemed medically fit for surgery
- Written informed consent

Exclusion criteria

- Unresectable disease/likelihood of R2 resection
- sMDT determined excess prior radiotherapy within IOERT target zone
- Women who are pregnant or breastfeeding
- Participation within an interventional clinical trial within 3 months of the point of registration within ELECTRA

RANDOMISATION PROCESS

Extensive pre-trial discussions and workshops with international stakeholders raised significant concerns about the potential for bias in such surgical trials. For example, it was considered that if the surgical team were aware in advance whether IOERT was to be delivered or not, that this may influence the radicality of surgery perhaps and extra margins may thus be taken in cases where IOERT may not have been applied, or in contrast, surgical margins may be reduced where it was known in advance that IOERT was to be delivered, thereby biasing outcomes and confounding interpretation. As a consequence of these discussions, blinding of the surgeon and oncologist was felt to be a critical bias-diminishing design introduced into ELECTRA.

Participants who meet the eligibility criteria for the study will be asked to consent to randomisation and blinding during the procedure to either no IOERT, standard dose IOERT (10Gy) or higher dose IOERT (15Gy).

During the procedure, the surgical team will conduct the exenterative procedure as intended and directed by the MDT determined surgical roadmap, and subsequently the IOERT team composed of a clinical oncologist, medical physicist, and radiographers are called to theatre. At this point the surgeon and clinical oncologist will assess the specimen, the tumour bed, and preoperative imaging and determine the utility of IOERT. Where IOERT is felt to be needed, the applicator is positioned, and the system set up.

The theatre is subsequently vacated (remote anaesthesia and monitoring is operational as standard) and patients will be randomised at this stage via a web-based system (1:1:1 ratio) by the lead physicist, who will be the only individual who will know the outcome of the randomisation, i.e., if IOERT will be administered and which dose, or if no IOERT is to be delivered. The surgeon, oncologist and patient will remain blinded throughout the study. Randomisation will be stratified by LARC or LRRC.

Blinding

The participant and clinicians (surgeons and oncologists) will not know the treatment allocation and will remain blinded throughout the study. Deaths and serious adverse events (SAEs) will be reviewed in a blinded manner.

Unblinding will be required in the event that any participant's further treatment may benefit from further radiotherapy, and if potential IOERT treatment and its dose may impact this.

Intervention and outcome assessments

IOERT

IOERT will take place in a purposely designed operating theatre with a portable self-shielded electron-beam linear accelerator, the MOBETRON (IntraOp Medical Corporation). The University Hospital Southampton (UHS) staff in surgery, oncology and radiography are fully trained in the use of the MOBETRON which has been in use since January 2017.

IOERT will be administered as determined to be best practice by the treating radiation oncologist, following previously described techniques in this patient group (19-24).

Oncological Outcomes

To assess the oncological outcomes the following data will be collected: IOERT field recurrence, overall local recurrence, and overall survival (at minimum 12 months post randomisation).

Cost effectiveness

The health economics aspect of the study aims to develop methods to collect resource use, cost and quality of life data to inform any future phase II/III study. It will:

• Develop methods to quantify resource use associated with the intervention (addition of IOERT and different doses to extended margin surgery), including complications.

• Explore changes in practice and resource use potentially attributable to the intervention by describing patients' treatment pathways with and without IOERT.

• Design and test the acceptability of a patient-completed questionnaire and nurse record diary to collect associated healthcare resource use.

• Investigate whether it is necessary to include the 5-level EuroQol 5-dimensions version (EQ-5D-5L) in the full study. Data will be collected using the preference-based EQ-5D-5L and disease-specific functional assessment of cancer therapy – locally recurrent rectal cancer quality of life (LRRC-QoL), 36-Item Short Form Survey Instrument (SF-36) and EORTC QLQ-C30 questionnaires at baseline and at each hospital visit. The acceptability and responsiveness of the questionnaires will be assessed, and EQ-5D-5L utility values (crosswalk and UK value set, if available at time of analysis) will be compared with

mapped utilities from LRRC-QoL, SF-36 and EORTC QLQ C30 calculated using validated algorithms from the literature.

Costs will be estimated for the UK National Health System (NHS). An NHS and social care perspective will be used, including intervention costs, outpatient visits and investigations, A&E attendances, hospital admissions, number and dose of each radiotherapy treatment. Itemised resource usage data will be priced using appropriate national sources: Personal Social Services Research Unit (PSSRU), NHS Reference costs and BNF (British National Formulary) for the UK.

The analysis of costs and quality of life will be descriptive and will include means and standard deviation (SD). Correlations will be used to assess evidence of sensitivity in the quality-of-life scores (EQ-5D-5L and mapped EORTC-QLQ-C30, SF-36 and LRRC-QoL) with the main outcome. The focus will be the direction of correlation and spread, and confidence intervals. A key post-op analysis parameter will be cases with a close or positive margin. Additional factors to be considered are complete/incomplete neoadjuvant course; previous pelvic radiotherapy; and dose of previous radiotherapy received.

Sample size

This is a feasibility trial, so the effectiveness of the intervention is not being evaluated; therefore, a formal power calculation was not deemed appropriate. The sample size is based on a 95% confidence interval approach, focused on estimating recruitment to the study. It is estimated that 80 eligible patients will be referred for consideration at the sMDT during the course of the study; this number ensures we will be able to estimate recruitment rate within 11%, sufficient to inform the planning of the future effectiveness study. Assuming just over 50% are eligible and agree to join the study, 42 participants will allow estimation of retention to within approximately 15%, as well as providing information on how IOERT is delivered.

Primary and secondary analyses

Analysis will focus on the endpoints relating to the feasibility of running a larger trial. The definitions of the primary objectives are given in Table 1 below. The primary analysis will be the frequencies and percentages for each primary endpoint, alongside 95%

confidence intervals (based on the Wilson interval). No hypothesis testing will be undertaken.

Secondary outcomes will be presented using suitable descriptive statistics according to the type of data, e.g., Kaplan-Meier (KM) plots, medians and 95% confidence intervals (derived from KM estimates for time-to-event data such as mortality). Summaries will be presented by randomised arm. Safety data will be presented by what (if any) dose was received – none, 10 Gy or 15 Gy. Where (non-time to event) data are collected at multiple time-points, summaries will be presented at each time-point. Missing data rates will be reported by arm as a percentage of those randomised to the arm.
